# Changes in heterosubtypic antibody responses during the first year of the 2009 A(H1N1) influenza pandemic

**DOI:** 10.1038/srep20385

**Published:** 2016-02-08

**Authors:** Gudrun S. Freidl, Henk-Jan van den Ham, Maciej F. Boni, Erwin de Bruin, Marion P.G. Koopmans

**Affiliations:** 1 Viroscience Department, Erasmus Medical Center, Rotterdam, the Netherlands; 2 Virology Department, Centre for Infectious Diseases Research, Diagnostics and Screening, National Institute for Public Health and the Environment, Bilthoven, the Netherlands; 3 Oxford University Clinical Research Unit, Wellcome Trust Major Overseas Programme, Ho Chi Minh City, Vietnam; 4 Centre for Tropical Medicine and Global Health, Nuffield Department of Medicine, University of Oxford, Oxford, UK

## Abstract

Seropositivity to avian influenza (AI) via low-level antibody titers has been reported in the general population and poultry-exposed individuals, raising the question whether these findings reflect true infection with AI or cross-reactivity. Here we investigated serological profiles against human and avian influenza viruses in the general population using a protein microarray platform. We hypothesized that higher antibody diversity across recent H1 and H3 influenza viruses would be associated with heterosubtypic reactivity to older pandemic- and AI viruses. We found significant heterogeneity in antibody profiles. Increased antibody diversity to seasonal influenza viruses was associated with low-level heterosubtypic antibodies to H9 and H7, but not to H5 AI virus. Individuals exposed to the recent 2009 A(H1N1) pandemic showed higher heterosubtypic reactivity. We show that there is a complex interplay between prior exposures to seasonal and recent pandemic influenza viruses and the development of heterosubtypic antibody reactivity to animal influenza viruses.

Influenza virus infection triggers the generation of antibodies as part of the humoral component of the host immune response. These antibodies, produced by specialized B-cells, are predominantly directed against the surface protein hemagglutinin (HA), and to a lesser extent, the neuraminidase (NA) and internal structures, such as the nucleoprotein and the matrix proteins^1^. HA and NA are used to classify influenza viruses into different subtypes. The 16 currently known HA-subtypes, originating from birds, divide into two phylogenetic groups based on their amino-acid composition, and these further segregate into 5 clades. Group 1 consists of three clades spanning ten HA-subtypes (H1, H2, H5, H6; H8, H9, H12; H11, H13, H16), whereas HA-subtypes H3, H4, H14 and H7, H10, H15 form the two clades of group 2 2,3. The HA consists of three monomers forming the variable globular head (HA1), which contains the receptor-binding site, and the more conserved stem region (HA2). The HA protein plays an important role in infection of host cells through the release of viral RNA into the host cell by means of membrane fusion^4^. Antibodies targeting influenza viruses can have neutralizing- or non-neutralizing ability. Non-neutralizing antibodies play a vital role in the immune response by e.g., inducing phagocytosis, complement-mediated lysis or antibody dependent cellular cytotoxicity (ADCC)^5^. Neutralization of influenza viruses can be achieved in two ways; either by blocking the receptor-binding pocket located in the HA1, or by preventing conformational changes in a region involved in membrane fusion, mainly formed by HA2^6^. The majority of antibodies target the HA1^7^. However, antibodies binding to the HA2 are able to neutralize various subtypes, reduce virus replication and contribute to a faster recovery^8^. Immunoglobulins targeting structures conserved among subtypes are termed as ‘cross-reactive’. A number of broadly reactive intra-subtype-, intra-clade-, intra-group- and inter-group specific neutralizing human and mouse monoclonal antibodies targeting the globular head- or the stem region of the HA have been identified (reviewed by Laursen and Wilson^9^). Their possible role in influenza virus infection has become an area of considerable interest since the occurrence of the most recent H1N1 influenza pandemic in 2009 [A(H1N1)pdm09]. Hancock *et al.*^10^ investigated whether seasonal, trivalent influenza vaccines are able to induce cross-reactive antibodies against the A(H1N1)pdm09 virus but did not find such antibodies after vaccination^10^. However, the authors reported on no or little pre-existing antibodies in individuals younger than 30 years of age, whereas in older adults some degree of neutralizing or cross-reactive antibody concentrations was detected in samples collected before the onset of A(H1N1)pdm09 circulation^10^. Wrammert *et al.*^11^ studied the serological response after natural infection with A(H1N1)pdm09 in humans and postulated that broadly cross-reactive antibodies targeting epitopes conserved between different influenza virus strains were induced via the activation memory B-cells. The detected antibodies predominantly targeted the HA2 and to a lesser extent HA1 of pre-pandemic H1 strains. Broadly H1N1-neutralizing antibodies also cross-reacted with avian subtype A(H5N1)^11^. These and subsequent studies showed that cross-reactive antibodies are boosted when infection occurs with a significantly mismatched HA^12^.

The effects of broadly-reactive influenza antibodies have not been studied extensively. Specifically, it is unknown if broadly reactive antibodies have any neutralizing effect during an avian influenza (AI) virus infection or if they generate false positive results in seroepidemiological studies on AI viruses. Zoonotic AI viruses pose a threat to public health; for instance, the highly pathogenic (HP) A(H5N1) subtype first crossed the species barrier into humans in 1997^13^,^14^. Since then, more than 800 human infections of A(H5N1) have been reported to the World Health Organization, of which 53% succumbed to the disease^15^. In recent years, additional HP and low pathogenic (LP) AIs have expanded the list of zoonotic subtypes causing incidental infection, e.g. LP H9N2, H6N2, H10N8, as well as various HP and LP H7 strains. Until recently, H7 strains were associated with mild symptoms in humans^16^ but in March 2013 a novel LPAI subtype (H7N9) emerged in China and has caused three waves of human infection associated with severe symptoms and a high case fatality rate^17^,^18^. Case fatality rates can be inflated if they only capture the most severe cases while mild or subclinical cases are underreported^19^,^20^. Sero-epidemiological studies are a useful way to shed light on the true extent of a population’s exposure to a particular virus. A number of serological studies have put forth evidence of human exposure to AIs in humans that work with animals^21^,^22^,^23^,^24^,^25^,^26^,^27^ as well as in putatively non-poultry-exposed control groups^28^,^29^. These findings pose the important question of whether serological reactivity against AI virus antigens reflects true exposure or is caused by cross-reactive antibodies.

In the present study, we investigated serological profiles against different human and AI virus subtypes during the course of the 2009 pandemic in a group of healthy childbearing age women via neonatal heelprick filter cards. Cards were collected continuously over a 100-week period employing a continuous collection study design^30^. Samples were analysed by means of a protein microarray comprising recombinant proteins representing the globular head domain (HA1) of various influenza virus HAs, as described previously^30^. Vaccination history of the mothers was unknown. Understanding serological profiles of healthy humans can help in distinguishing heterosubtypic antibody reactivity from serological response triggered by true infection.

Here, we hypothesized that the profile of antibody reactivities to a range of recent human influenza viruses could be used to explain the presence of cross-reactive antibodies to AI antigens (H5, H7 and H9). We found evidence that supported this hypothesis and showed that cross-reactive antibody levels to AI and ancient influenza virus subtypes significantly increased after the onset of the 2009 H1N1 pandemic.

## Results

### Exploratory analysis

Characteristics of the study population are shown in Table 1. The majority of samples collected through the heel prick-screening program were submitted from countries located in the northern hemisphere (n = 6896), with only 688 samples collected in the southern hemisphere. Submission periods differed per country and covered the time span from week 40 in 2008 to week 34 in 2010 (Table 1).

**Figure 1:**
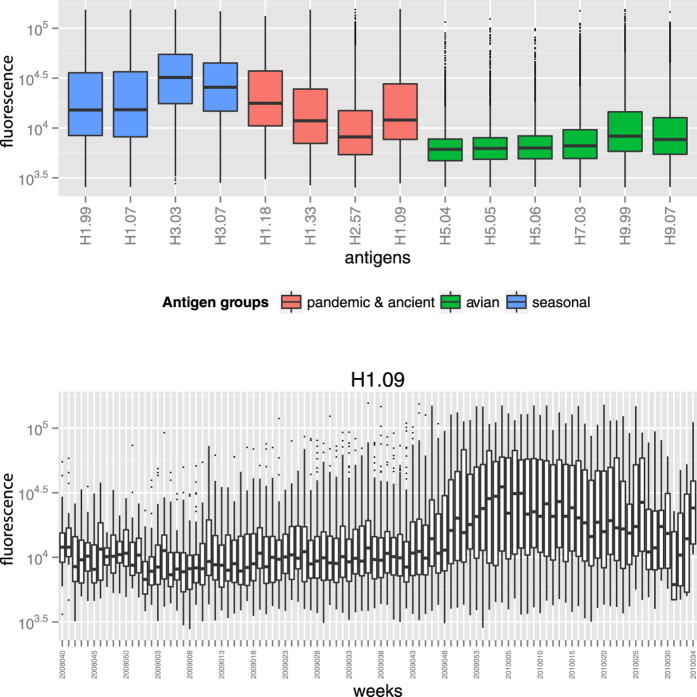
(**a**) Overall antibody reactivity against different antigens for the entire study period (week 40, 2008 to week 34, 2010) including all countries. (**b**) Development of A(H1N1)pdm09 over time for all countries combined. Pandemic onset and -course per country were previously described in de Bruin *et al.*^30^. Both y-axes represent fluorescence values on a log10-scale.

The highest antibody levels were directed to seasonal H1 and H3 antigens, as expected (Fig. 1a, blue). Elevated signals against antigen H1.09 were observed (Fig. 1b), and their levels were clearly associated with the onset of the pandemic in the second half of the study (Fig. 1b). Similarly, raised signals against 1918-lineage influenza strains (H1.18, H1.33) were associated with pandemic onset (see supplementary material S1), as the H1.18 antigen is known to be antigenically similar to A(H1N1)pdm09^31^. In 1957 subtype H2N2 (represented by antigen H2.57) caused the second major human pandemic of the 20th century. In 1968 – before the mothers of our study subjects were born – H2N2 ceased circulating in the human population^32^. Nevertheless, antibody signals to this antigen were raised, albeit at significantly lower levels compared to reactivity against recent H1 and H3 antigens (Fig. 1a, red; Wilcoxon signed rank test, p-values <0.001). Antibody signals to H9.99 were similarly raised as those against H2.57 (Wilcoxon signed rank test: p-value = 0.15). Fluorescence levels against H9.07 and H7.03, although also elevated, were significantly lower compared to H2.57 (Wilcoxon signed rank test, p-values <0.001). Reactivity against these AI antigens was most likely caused by cross-reactive antibodies^33^,^34^. No noteworthy reactivity against H5-antigens was found (Fig. 1a, green).

### Antibody diversity across human influenza HA1 antigens and its relation with heterosubtypic reactivity

**Figure 2:**
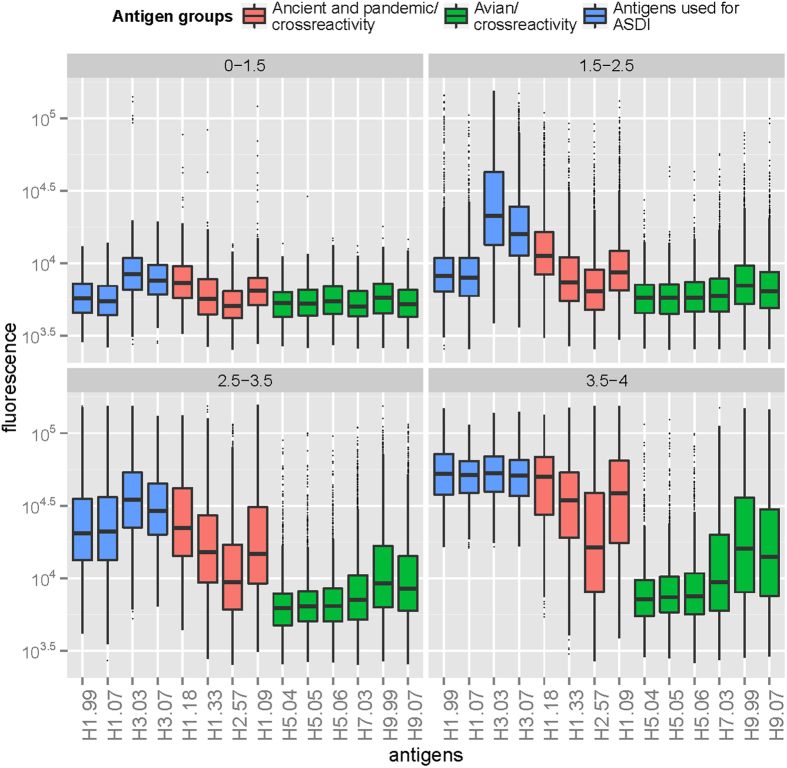
Serological profiles based on adapted Shannon diversity index (ASDI) categories. Recent seasonal influenza virus antigens were used to calculate ASDI per individual to summarize individual antibody profiles in one measure (blue). Assumed cross-reactive antibody responses are depicted in red (ancient- and older pandemic influenza virus strains) and green (avian influenza virus strains). Fluorescence values representing serological reactivity per antigen (x-axis) are shown on a log10-transformed y-axis.

To examine antibody diversity in our study subjects, we introduced the adapted Shannon diversity index (ASDI; see Methods and supplementary material), which aims to represent the number of antigens to which an individual has a high titer response. The influenza antigens included in the ASDI measure represented recent seasonal influenza viruses H1.99, H1.07, H3.03 and H3.07. ASDI values were calculated per individual and ranged from 0.64 to 4.0, with a value of 4.0 meaning that the individual had high titer responses to all four antigens in the ASDI measurement. We arbitrarily divided the ASDI range into four categories to assess corresponding serological profiles and investigate the association between heterosubtypic responses and increasing ASDI. The majority (~77%) of individuals had antibody signals to 1.5 to 3.5 antigens (ASDI categories 2&3; Table 1). The category with lowest diversity (0–1.5; n = 416) was characterized by the lowest level of H3 responses when compared to other ASDI categories and comprised slightly raised signals to H1.18 and H1.09 antigens (Fig. 2). A similar pattern was observed for category two (1.5–2.5; n = 2548) with predominant seasonal H3 signals, together with somewhat elevated seasonal and pandemic H1 responses. We also observed high H3 responses in the third category (2.5–3.5; n = 3272), albeit in combination with markedly increased H1 signals compared to lower categories. The fourth category (3.5–4; n = 1348) comprised individuals with the highest antibody diversity and reactivity against H1.09. Pandemic, seasonal H1, and seasonal H3 antigens were approximately equally strong in this group, partly reflecting saturated luminescence signals in the assay. A total of 161 individuals (2.1% of the total population; 0.8% and 9.9% of diversity category 3 and 4, respectively) had saturated fluorescence values for H1.09 and all four seasonal antigens; all but one of these individuals were sampled after pandemic onset.

### Analysis of Broad responders

Based on the high and broad signals in the fourth category (3.5 <ASDI ≤4.0), we designated subjects in this category as ‘broad responders’, i.e. individuals showing serological responses to between 3.5 and 4 antigens. Approximately half of the individuals in the fourth category (52%, n = 702) were from the northern hemisphere (Canada, USA, Sweden, United Kingdom and Japan) and had been sampled after pandemic onset (Table 1).

Consistent with our hypothesis, we observed a gradual elevation in heterosubtypic reactivity against avian influenza antigens with increasing diversity categories (Fig. 2, green). Broad responders (3.5 <SDI ≤4.0) showed the highest reactivity against avian antigens, which was most pronounced for H9, followed by H7; reactivity to H5 antigens remained low (Fig. 2, green). However, some cross reactivity was found in each diversity category, and we found statistically significant Spearman’s rank correlation coefficients between diversity indices and AI HA1 reactivities (0.25–0.29 for H5, 0.35 for H7 and 0.42–0.43 for H9; all p < 10^^−15^). Similarly, some heterosubtypic reactivity against ancient and older pandemic human strains (H1.33 and H2.57) was detected with increasing diversity categories (Fig. 2, red).

### Explaining heterosubtypic reactivity by serological responses to recent seasonal and pandemic strains

We performed a multivariable linear regression analysis using reactivity to human seasonal and recent pandemic influenza HA1 antigens as explanatory variables to explore the relationship between serological responses to AI antigens and infection with recent human influenza virus strains (Table 2). Antibody reactivities to most included antigens (i.e. explanatory variables) were able to explain the variation observed in H7- and H9 signals to some extent (Table 2), as these reactivities predominantly tended to be positively correlated (Table 2). However, antibody reactivities to the most recent human influenza strains H1.09 and H3.07 had a larger relative effect on the variation of H7 and H9 signals compared to signals against other antigens, indicating that recent infection with these viruses can explain part of the low-level heterosubtypic antibody reactivity to AI virus antigens. Nevertheless, the models could only explain between 28% and 38% of observed variation in H7 and H9 avian responses, suggesting a more complex relationship (Table 2). Multicollinearity between explanatory variables was not an issue as variance inflation factors for all explanatory variables in all models remained below 10 (range: 1.41–3.59). Likewise, testing model assumptions revealed no overt violations of homoscedasticity and deviation from normality of residuals after log-transformation. Regression analysis was not attempted for H5, given the low antibody signals.

### The influence of A(H1N1)pdm09 on the level of cross-reactive antibodies

To further examine whether exposure to the novel pandemic influenza strain A(H1N1)pdm09 was associated with increased cross-reactivity, we divided the data set into two periods of before (n = 3337) and after pandemic onset (n = 4247) (Table 1). We observed a clear shift in proportion of broad responders (category 4) towards higher diversity categories after pandemic onset (Chi-squared test, p-value <0.001) (Table 3a). Proportions of H1.09-seropositive individuals gradually increased with increasing diversity index categories for pre- and post-pandemic periods. Within each category, changes in proportions between pandemic periods according to seropositivity status were significant for all but the lowest category, which could not be tested due to too few observations (Chi-squared test, p-values <0.001, Table 3b). The vast majority of H1.09-seropositive individuals were sampled in the post-pandemic period (3.3% pre-, versus 29% post pandemic, Table 3b).

**Figure 3:**
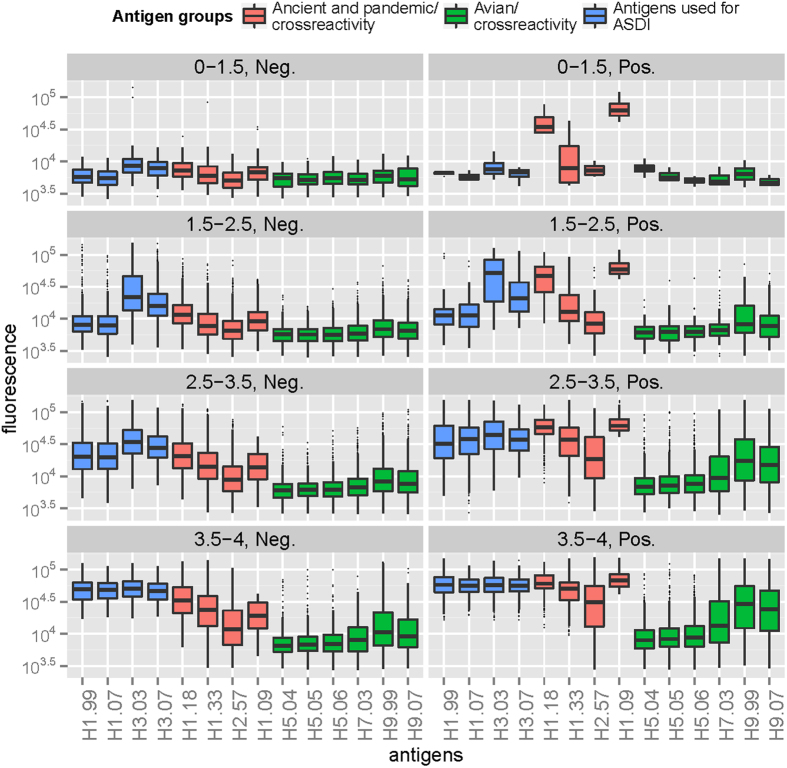
Antibody profiles of samples collected after pandemic onset, stratified according to adapted Shannon diversity index (ASDI) and seropositivity status to A(H1N1)pdm09.

Next, we only included data from persons sampled after the pandemic onset in the analysis, to ensure that serological responses were truly triggered by H1.09 infection or vaccination (Table 3b). With exception of the lowest ASDI category that only comprised four individuals in the H1.09 positive category (Table 3b), within higher ASDI categories, we generally observed higher heterosubtypic antibody responses among H1.09-positive individuals compared to negative ones (Fig. 3). Post-pandemic onset, 57% of broad responders (highest diversity category 4) were seropositive for H1.09 (Table 3b). In this category, we also observed significantly higher levels of H7 and H9 antibodies compared to H1.09-negative persons (Fig. 3; Wilcoxon rank sum test, p-value < 0.001).

### Heterosubtypic reactivity and its consequences of for seroprevalence studies of avian influenza in humans

**Figure 4:**
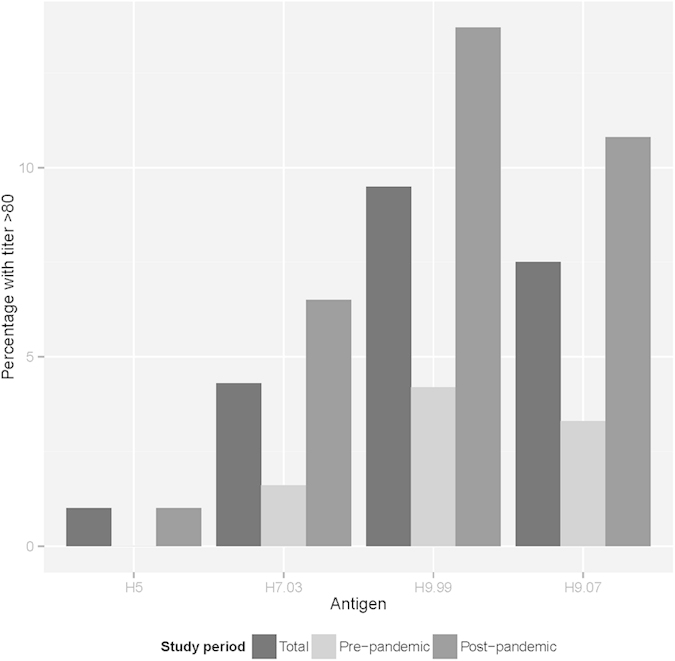
Estimated proportions of individuals with titers to avian influenza virus antigens of higher than approximately 80. Proportions are presented for the total study period and split according to pre- and post pandemic periods. Reactivity to the three H5 antigens is combined.

When estimating the proportion of individuals with titers of approximately higher than 80 against AI antigens (based on an arbitrary cut-off applied to fluorescence values as described in the Methods section), we found that overall, about 1% (n = 53–80) of individuals had antibody titers >80 for H5 antigens, and 4.3% (n = 329), 9.5% (n = 720) and 7.5% (n = 571) of individuals showed titers >80 to H7.03, H9.99 and H9.07, respectively. The majority thereof were sampled in the post-pandemic period [1% (n = 49–76), 6.5% (n = 276), 13.7% (n = 580) and 10.8% (n = 460), respectively, Fig. 4].

## Discussion

In this study, we investigated serological profiles against human influenza virus subtypes in healthy humans from around the world and studied associations between serological responses to seasonal influenza viruses and heterosubtypic reactivity, defined as presence of antibodies to influenza viruses that have not circulated among humans. A validated microarray platform^35^ comprising recombinant HA1 proteins, i.e. the globular head of the HA, was used to provide standardized serological profiles to a range of influenza A virus antigens in this population-based study.

The highest antibody titers were observed against H3-virus antigens [A(H3.03), A(H3.07)]. This subtype emerged in the human population in 1968, and in 1977, subtype A(H1N1) re-emerged and co-circulated with A(H3N2) until 2009^32^. Given the assumed range of birth years of our study population (between 1968 and 1990), a significant proportion of individuals was probably primed (i.e. experienced their first influenza infection) by subtype A(H3N2) influenza viruses. These findings are consistent with the observation of original antigenic sin^36^,^37^, a phenomenon by which an individual’s first influenza infection imprints a high life-long specific antibody titer in that individual. Under this hypothesis, infections with recent strains thereby serve as a booster for antibodies to the ‘original’ strain, whereas specific antibodies to the recent strain itself may be detected at low or moderate levels.

A number of serosurveillance studies conducted on high risk groups revealed serological evidence for avian viruses^23^,^24^,^25^,^38^,^39^,^40^, although the possibility of cross-reactive antibodies for most studies was raised in a commentary article^41^. The level of seropositivity may also be influenced by the type of assay and respective cut off levels used (e.g. hemagglutination inhibition- (HI), microneutralization (MN)- or pseudotype-based assay)^42^. In this study, we investigated cross-reactive antibodies to AI virus antigens in the general population, and its relation to antibody diversity against recent human influenza virus strains by developing an adapted Shannon diversity index (ASDI) as a summary measure describing both antibody diversity and total antibody concentration. Consistent with our hypothesis, we demonstrated a positive association between increasing antibody diversity and heterosubtypic reactivity against AIV antigens. These observations are consistent with previous publications that have found raised antibody titers to AI antigens in the general population^28^,^29^,^30^,^31^,^32^,^33^,^34^,^35^,^36^,^37^,^38^,^39^,^40^,^41^,^42^,^43^,^44^. A population-based studies on heterosubtypic immunity of intravenous immunoglobulins (IVIG) from blood donors from Australia, Malaysia and Europe clearly showed binding of heterosubtypic antibodies against H9 and H5, but negligible binding against H7 subtypes by immunoblotting. In IVIG formulations from all regions neutralizing ability could be confirmed for H5 subtypes using cell culture^45^. Consistent with our findings, a study conducted in the general population from rural and urban locations in Vietnam, using the same protein microarray, reported similarly elevated antibody titers to H9 and to a lower extent H7 and H5 antigens^33^. Similarly, depending on the cut-off used (ranging from 80 to 20), 0.25% to 9.4% and 1.8% (cut-off of 20) of the general population in Wuhan, China, tested seropositive for H9 and H7 by HI-assay, respectively^43^. Serological evidence for antibodies to influenza A(H5N1) was also detected in the Italian general population by single radial hemolysis, but could not be confirmed by HI- or MN-assay^44^.

With the onset of the A(H1N1) pandemic, pre-existing immunity and heterosubtypic antibody responses to AI viruses in the general population became of significant interest^10^,^11^,^12^. For this reason, we focussed our analysis of cross-reactive antibody patterns on this cohort, to perform inference on AI antibody concentrations only, and to describe associations with other covariates. During the unfolding of the pandemic, overall cross-reactive responses to AI viruses increased. Multiple linear regression analysis suggested that H1.09 responses could explain part of this heterosubtypic reactivity but there was considerable heterogeneity in antibody profiles, with persons responding differently to similar challenges, most likely due to differences in exposure history. Therefore, the history of exposures to human seasonal and pandemic influenza exposures, natural or vaccine-induced, can influence levels of antibodies that bind to animal viruses^46^.

The present study design had some limitations that need to be weighed when looking at the effects of human influenza infections on heterosubtypic antibody response. First, we were unable to discern whether serological reactivity against H1.09 was triggered by natural infection or by vaccination with the novel pandemic strain, as the onset of circulation of A(H1N1) pdm09 coincided with the beginning of vaccination campaigns in the majority of participating countries^30^. A recent study in pregnant women demonstrated that immune response elicited by vaccination to A(H1N1)pdm09 was significantly higher than after natural infection. This observation was also reflected in newborns of vaccinated mothers, with 89.5% showing antibodies to the pandemic strain, compared to 15.8% of infants born to naturally infected mothers^47^. Second, a limitation is that all study participants were of childbearing age. Although this subset of the general population represents an unbiased sample, extrapolation of these conclusions to the general population, including young infants and older age groups, should be made with caution. The assumption that our study participants were between 20 and 40 years of age, seemed appropriate. Estimated mean ages of women at childbearing ranged from 26.3 to 30.9 between 2005 and 2010 for the participating countries^48^. However, we cannot fully exclude that a small proportion of individuals was older and experienced natural infection with H2N2, a subtype that may generate additional cross-reaction to AI. Furthermore, no information on poultry exposure was available. Given the near global distribution of AI, we cannot exclude that part of the study population might have been exposed to and possibly infected with AI viruses. However, only five participating countries (the Netherlands, USA, Canada, India, Japan) reported AI outbreaks (H5, H7) in birds during the period of our study^49^.

Finally, whereas the microarray platform serves as an excellent screening tool to investigate population exposure to various influenza virus HA1s in a standardized fashion, it cannot provide information on functionality of heterosubtypic antibodies against avian antigens detected in our study (i.e. neutralizing ability), neither can it measure cross-reactive antibodies against the HA2 stem region of the HA. Previous experiments using the entire recombinant HAs of different subtypes in the microarray platform had low discriminatory ability, as the HA2 is more conserved between different influenza virus subtypes. Using HA1s, therefore, provides a better resolution of antibodies targeting the more variable globular head of the HA, thereby allowing subtype discrimination. Although we cannot directly generalize antibody reactivities against the HA1 to the entire HA, we previously showed good correlation between antibody titers measured by HI and by microarray HA1 proteins^33^,^34^,^35^. Longitudinal studies examining heterosubtypic responses using functional assays would shed light on this issue. This is in fact the major limitation in all human serological studies of AI - it is unknown whether assay results correlate to any level of severity or protection from infection. Given the pandemic potential of AI virus subtypes, investigating the protective effect of cross-reactive responses to AI viruses in the general population would aid pandemic preparedness by providing information on herd immunity^50^. Ascertaining, whether antibody responses against avian influenza viruses reflect true exposure or cross-reactivity, remains a challenge. To address this issue further, establishing antibody profiles from humans exposed to avian influenza viruses during AI outbreaks could be considered, while also systematically expanding population-level serological studies by including exposure and vaccination history. Such an approach would allow studying kinetics of low-level heterosubtypic antibody responses and comparison of serological profiles in high- versus low risk populations, thereby potentially aiding unbiased interpretation of such findings. For these purposes, the microarray platform could serve as a broad first screening assay, which could be followed by additional serological tests, such as the HI- or MN-assay to ascertain functionality of the detected antibodies.

## Methods

### Study population

In a study conducted to monitor the progression of the A(H1N1)pdm09 in different parts of the world, 13 countries from five continents contributed more than 7000 anonymized, filter cards containing dried blot spots from heel prick sampling^30^ (Table 1) through neonatal screening programs. This collection method, originally implemented to test for hereditary diseases in new-borns, can also be used to measure maternal antibodies conferred via the placenta^51^. As samples could only be collected when anonymized, we assumed the age of the mothers to be between 20 to 40 years, translating to birth years ranging from 1968 to 1990. We furthermore hypothesized that study subjects in our data set reflect a segment of the general population with unknown prior poultry exposure, thus providing an unbiased systematic population sample.

### Ethical approval

As previously described in de Bruin *et al.*^30^, participants were included in the study in accordance with local medical ethical rules. Samples were collected within neonatal screening programs and parents provided informed consent for using residual samples (anonymized) for research purposes. The study was approved by the Japanese Institutional Review Board of the Sapporo City Institute of Public Health (reference number 09-010) and the American NYS DOH Institutional Review board (protocol number #09-045). Participating laboratories collected 10 randomly selected anonymized filter paper cards per week, concordant with regulations of local ethical committees.

### Protein microarray technique

IgG levels against different human and avian influenza HA types were measured using a protein microarray platform as described previously^30^,^31^,^32^,^33^,^34^,^35^. Briefly, recombinant proteins of the HA1 part of HA of different influenza virus subtypes (see supplementary material S2) were printed onto nitrocellulose-coated glass slides (64pad, Oncyte Avid, Grace Biolabs, Bend, USA) using a non-contact Piezorray spotter (Perkin Elmer, Waltham, USA). Subsequently, dried blood spots were eluted as described previously and samples were tested at a 1:80 dilution^30^. A Dylight649-labelled goat-anti-human IgG (Fc-fragment specific, Jackson ImmunoResearch) was used to bind to serum antibodies and fluorescence was quantified by means of a microarray scanner (ScanArray, Perkin Elmer). The protein microarray technique allows simultaneous and standardized detection of antibodies against different influenza subtypes in a minute serum quantity. It has also been used to measure influenza IgG titers in humans^35^.

### Data analysis

Data analysis was performed in R (version 3.1.0, R Statistical Computing, Vienna, Austria). For all statistical analyses, a p-value of less than 0.05 was considered statistically significant. All samples were normalized to a mean background fluorescence of 5000. Correction for day-to-day variation between microarray slides was achieved based on H1.09 signals against an international standard positive control as described before^30^.

For exploratory data analysis, overall fluorescence values between different antigens were compared using the Wilcoxon signed rank test. To characterize antibody profiles and study possible profile-specific heterosubtypic reactivities against AI antigens, we summarized individual serological responses against multiple antigens using the Shannon diversity index, which is a measure frequently applied in ecological studies to quantify biodiversity of species within habitats (see supplementary material S3). For our purpose, we adapted the Shannon diversity index (ASDI) so that we could detect both diversity and magnitude of an antibody response (a traditional Shannon diversity index only describes magnitude). To achieve this effect, we included a dummy serological response with a fluorescence value of 50.000 to ensure that low-and-broad antibody profiles receive a low ASDI score (see supplementary material S3). Only seasonal human influenza antigens that circulated in the 10 years prior to the sample collection were included in the ASDI calculation (H1.99, H1.07, H3.03, H3.07; Table S1). The most recent pandemic strain H1.09 was not included in the ASDI calculation, so that we could investigate its effect on antibody profiles within ASDI categories. The dummy strain’s contribution was subtracted off, and the ASDI can thus be thought of as the number of strains to which an individual has a high antibody response; the maximum diversity being 4.0. For the purposed of presentation, we divided the ASDI range into four arbitrary categories ranging from ‘0–1.5’, ‘1.5–2.5’, ‘2.5–3.5’, and ‘3.5–4’. Associations between ASDI and avian fluorescence signals were evaluated using a Spearman correlation coefficient.

We used R packages ‘psych’^52^ and ‘ggplot2’^53^ for exploratory analysis and to create figures, respectively. Package ‘MASS’^54^ was used for a multivariable log-log linear regression model (with backward elimination using function ‘stepAIC’) to investigate whether antibody reactivity against recent human antigens (H1.99, H1.07, H3.03, H3.07, H1.09) could explain serological reactivity against avian antigens. Repeating the analysis using a ‘forward selection’ algorithm yielded the same results. The full model included all recent human H1 and H3 antigens as explanatory variables. Final models included only significant explanatory variables presented in this table. R package ‘car’^55^ was used to calculate the variance inflation factor to check for multicollinearity between explanatory variables.

For comparison of mean ranks of fluorescence levels per antigen during the A(H1N1) pandemic of 2009/2010, we used the non-parametric Wilcoxon Rank-Sum test with continuity correction on data stratified by pre- and post-pandemic sampling periods. For this, “time of pandemic onset” was selected based on country-specific pandemic curves as shown in de Bruin *et al.*^30^ (Table 1). To determine a cut-off for the pandemic H1.09 HA1 antigen signals, we used H1.09 data of samples collected before the official onset of the pandemic in April 2009. The cut-off was established using mean fluorescence levels plus three standard deviations. Using this cut-off, we reported proportions of H1.09-seropositive individuals within ASDI categories.

To investigate the effect of the A(H1N1)pdm09 on the proportions of broad responders, a Pearson’s chi-squared test was used to test for changes in proportions before and after pandemic onset.

As samples of the study population were tested in a one-point dilution (1:80), we used this arbitrary cut off to approximate proportions of individuals with an estimated antibody titer of higher than 80 to avian antigens. Based on prior studies^35^, a fluorescence cut-off point of ~30.000 corresponds to an antibody titer of higher than approximately 80.

## Additional Information

**How to cite this article**: Freidl, G. S. *et al.* Changes in heterosubtypic antibody responses during the first year of the 2009 A(H1N1) influenza pandemic. *Sci. Rep.* **6**, 20385; doi: 10.1038/srep20385 (2016).

## Supplementary Material

Supplementary Information

## Figures and Tables

**Table 1:** Characteristics of study population and number of samples submitted per country and time period.

Continent	Country	Country totals	Row totals	Pandemic onset^1,2^	Sampling period (year/week)	Diversity index categories			
						1	2	3	4
						0–1.5	1.5–2.5	2.5–3.5	3.5–4
North America	Canada	913	444	Pre	08/40–09/32	6.8	34.7	45	13.5
			469	Post	09/33–10/26	1.1	17.1	48	33.9
	Mexico (central)	579	272	Pre	09/26–09/45	21	40.4	32.7	6.2
			307	Post	09/46–10/24	8.1	41.7	36.2	14
	Mexico (northern)	432	432	Pre	09/01–09/044	18.8	50.9	26.6	3.7
			0	Post	NA				
	USA	520	130	Pre	09/28–09/40	0.8	24.6	47.7	26.9
			390	Post	09/41–10/26	0.3	8.7	38.7	52.3
Europe	The Netherlands	559	188	Pre	09/27–09/45	1.6	46.8	40.4	11.2
			371	Post	09/46–10/30	0.5	32.1	50.1	17.3
	Portugal	479	130	Pre	09/28–09/40	13.8	54.6	28.5	3.1
			349	Post	09/41–10/23	5.2	33.8	44.4	16.6
	Sweden	868	526	Pre	08/40–09/40	2.5	46.4	44.3	6.8
			342	Post	09/41–10/22	0.3	20.2	49.1	30.4
	Switzerland	637	180	Pre	09/23–09/40	3.3	31.1	52.2	13.3
			457	Post	09/41–10/34	3.5	33.5	50.3	12.7
	UK	568	190	Pre	09/27–09/45	7.9	39.5	40	12.6
			378	Post	09/46–10/30	2.6	27.5	47.4	22.5
Asia	India	474	120	Pre	09/28–09/40	4.2	31.7	51.7	12.5
			354	Post	09/41–10/25	3.4	34.5	48.3	13.8
	Japan	530	140	Pre	09/27–09/40	1.4	21.4	51.4	25.7
			390	Post	09/41–10/26	0.3	13.6	47.7	38.5
	Lebanon	337	337	Pre	09/02–09/44	11.3	49.3	34.4	5
				Post	NA				
Africa	South Africa	276	248	Pre	09/29–10/15	12.1	48	35.1	4.8
			28	Post	10/16–10/21		42.9	42.9	14.3
South America	Argentina	412		Pre	NA				
			412	Post	09/27–10/16	6.6	37.1	43.4	12.9
Total range of study weeks:					08/40–10/34				
Totals per pandemic period	pre: 3337	post: 4247							
Totals per diversity category						416	2548	3272	1384
Total									7584

^1^Pandemic onset of A(H1N1)pdm09; ^2^pre- and post pandemic onset of circulation of A(H1N1)pdm09 per respective country, percentages are represented within pandemic period; NA: not available.

Diversity index categories reflect percentage of individuals within the respective category.

**Table 2:** Regression coefficients calculated on log2-transformed data spanning pre- and post-pandemic periods.

Outcome	Intercept	Estimates with standard errors (SE)					AdjustedR2
		H1.99	H3.03	H1.07	H3.07	H1.09	
**H7.03**	5.99	-0.03	0.05	0.05	0.16	0.25	0.28
SE	0.14	0.013	0.011	0.013	0.012	0.009	
	***	*	***	***	***	***	
**H9.99**	3.90	-0.12		0.14	0.29	0.35	0.38
SE	0.15	0.014		0.014	0.011	0.010	
	***	***		***	***	***	
**H9.07**	4.22	-0.05	0.08	0.11	0.16	0.32	0.37
SE	0.15	0.013	0.011	0.014	0.013	0.011	
	***	***	***	***	***	***	

Outcome refers to reactivity against heterologous antigens on which serological responses against recent human influenza virus antigens were regressed (explanatory variables). The number of asterisks indicates level of significance.

Significance codes: 0 ***0.001 **0.01 *0.05.1.

**Table 3:**
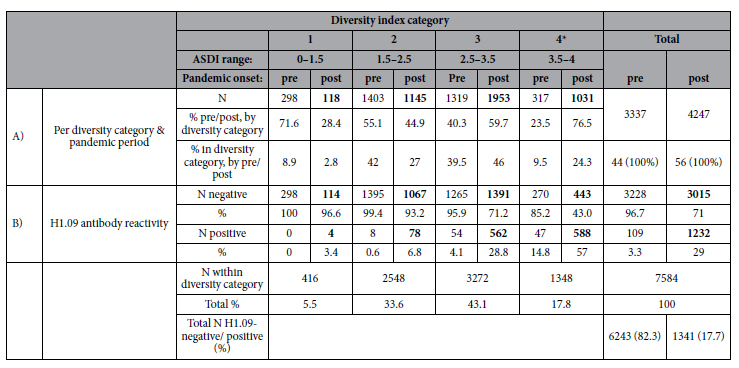


(**A**) Number and proportions of individuals per category based on adapted Shannon diversity index (ASDI) versus pre- and post-pandemic periods. (**B**) Number and proportion of H1.09-positive and -negative individuals per diversity category before (n = 3337) and after (n = 4247) pandemic onset, respectively.

*Category of ‘Broad responders’, defined as showing the highest antibody diversity across recent seasonal human influenza viruses (H1.99, H1.07, H3.03, H3.07), expressed by the Adapted Shannon diversity indices (ASDI).
