# Supplementary material for: Changes in heterosubtypic antibody responses during the first year of the 2009 A(H1N1) influenza pandemic
Source: Sci Rep. 2016 Feb 8;6:20385. doi: 10.1038/srep20385 (PMC4745054; doi:10.1038/srep20385)
Supplement: Supplementary Information [file srep20385-s1.pdf]

Supplementary material to:

Changes in heterosubtypic antibody responses during the first year of the 2009 A(H1N1) influenza pandemic

Gudrun S. Freidl [1,2*], Henk-Jan van den Ham[1], Maciej F. Boni [3,4], Erwin de Bruin[1,2] and Marion P.G. Koopmans[1,2]

[1]Viroscience Department, Erasmus Medical Center, Rotterdam, the Netherlands

[2]Virology Department, Centre for Infectious Diseases Research, Diagnostics and Screening, National Institute for Public Health and the Environment, Bilthoven, the Netherlands

[3]Oxford University Clinical Research Unit, Wellcome Trust Major Overseas Programme, Ho Chi Minh City, Vietnam

[4]Centre for Tropical Medicine and Global Health, Nuffield Department of Medicine, University of Oxford, Oxford, UK

*Corresponding author: gudrun.freidl@rivm.nl

S1. Serological responses to H1.18 and H1.33 were associated with the development of the H1N1 2009 pandemic in the second half of the study. Fluorescence values are depicted over the entire study period (expressed in weeks on the x-axis) for all countries combined. Y-axes represent fluorescence values on a log10-scale.

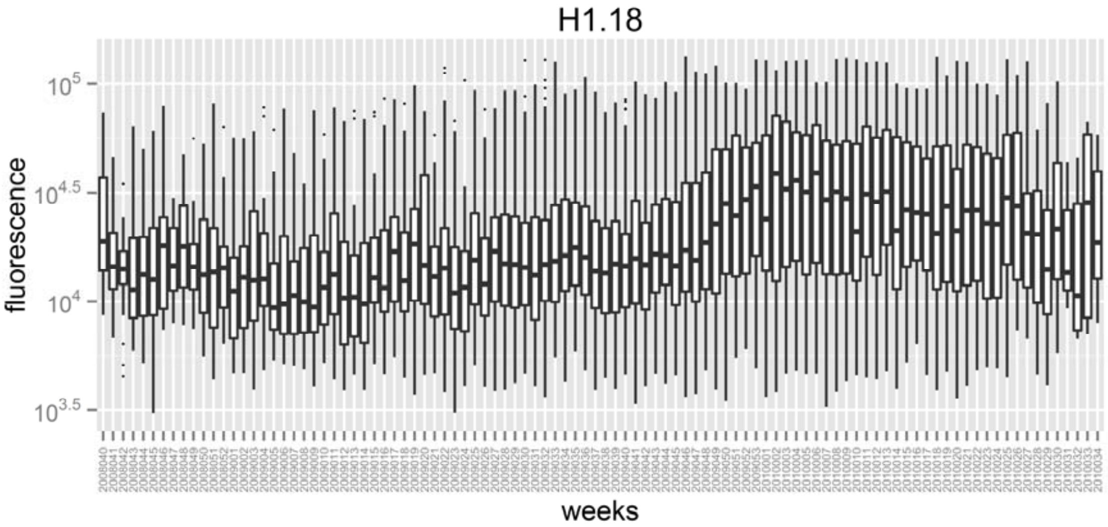

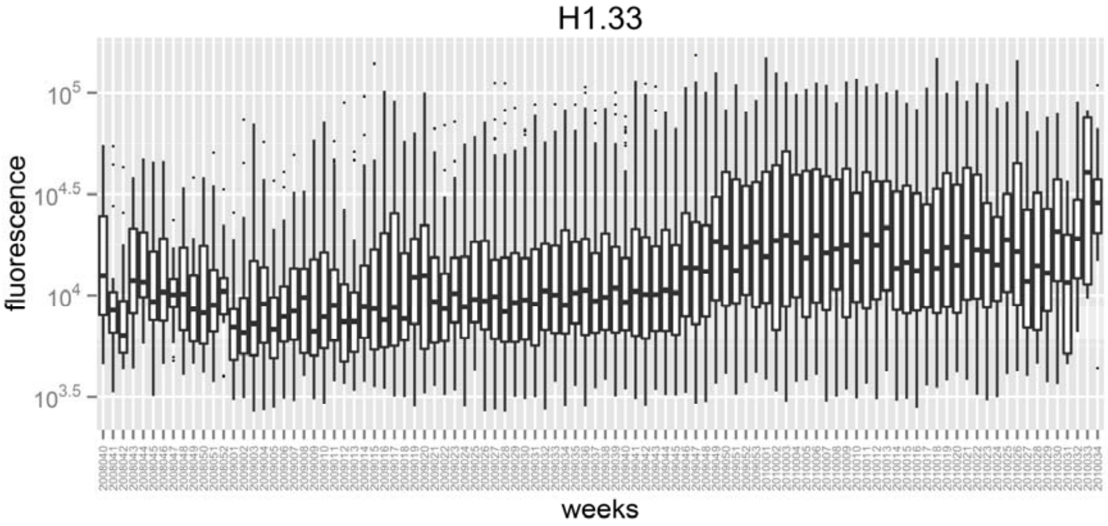

S2. Table depicting recombinant proteins used for the production of protein microarray slides used in this study [1].

Antigen	Influenza virus strain	Manufacturer
H1.18	A/South Carolina/1/18	Immune Technology Corp
H1.33	A/WS/33	Immune Technology Corp
H1.99	A/New Caledonia/20/99	Immune Technology Corp
H1.07	A/Brisbane/59/2007	Immune Technology Corp
H1.09	A/California/6/2009	Immune Technology Corp
H2.57	A/Canada/720/05*	Immune Technology Corp
H3.03	A/Wyoming/3/03	Immune Technology Corp
H3.07	A/Brisbane/10/2007	Immune Technology Corp
H5.04	A/Vietnam/1194/2004	Immune Technology Corp
H5.06	A/Turkey/15/2006	Genscript
H5.05	A/Indonesia/5/2005(H5N1)	Genscript
H7.03	A/Chicken/Netherlands/1/03	Immune Technology Corp
H9.99	A/Guinea fowl/Hong Kong/WF10/99	Immune Technology Corp
H9.07	A/Chicken/Yunnan/YA114/2007	Genscript

*Influenza virus strain of subtype H2 was isolated in 2005 in course of a laboratory accident

S3. Adapted Shannon Diversity index (ASDI)

There are several different diversity measures. Each of them has a different range and all their values have different interpretations. In general, these values can be represented as the number of responses of equal magnitude so as to provide an intuitive measure that everyone may understand. Furthermore, this approach collapses many diversity measures onto each other, since they lead to the same species diverity when mapped back to the number of equally-abundant species (for details, see [2]).

The key to this approach is the transformation of the diversity measures to species:

$$^{q}D = \left(\sum_{\substack{S \\ i=1}} (p_i)^q \right)^{\frac{1}{1-q}}$$

where *D* is the diversity measure of order *q*, *S* is the number of species, and

$$p_i = \frac{\{s_i\}}{\sum_i^S s_i}$$

i.e., the fraction of each species in the data, where s_i is the set of $s_1, s_2, \ldots$.
The interpretation of this index is diversity equivalent to that number of equally abundant species. For instance, a diversity of 3 means: 3 equally abundant species.

Adaptation to antibody measurements:

The index is adapted to be used for evaluating antibody measurements, i.e., fluorescence. To quantify diversity of serological responses, this index can be used. However, to down-weigh the influence of low fluorescence values, we included a parameter that sets the minimal level of what is considered an effective response. By adding an element to s_i, the index has an offset or *anchor* to distinguish between background and real responses:

$$^{q}D' = \left(\sum_{\substack{S \\ i=1}} (p'_i)^q \right)^{\frac{1}{1-q}} - 1$$

where

$$p'_i = \frac{\{k, s_i\}}{k + \sum_i^S s_i}$$

with

$$k = \max(k_0, s_i)$$

Properties:

For $\max(s_i) \sim k_0$, $qD' \rightarrow qD$.

For $\max(s_i) \ll k_0$, $qD' \rightarrow 0$.

NB: Please note that the anchor is not a cut-off, as the values below it are given less weight, but are still taken into account.

Implementation in R

```
qDp = function(values, q=2, cutoff = 0, offset=50000){

 ## the offset prevents patients with all-round low values
 ## from having high diversity scores

 ## prevent the offset from
 ## pulling down the diversity score when high
 if(max(values)>offset) offset = max(values)

 values = c(values, offset)
 pp = values/sum(values)
 Div = (sum(pp^q))^(1/(1-q))

 return(Div-1) ## subtract 1 to remove the offset again.
}
```

Examples of diversity scores

Some examples of diversity scores are included below. The grey bars indicate antibody fluorescence values to different antigens, the blue bar indicates the anchor. The maximum diversity is the number of grey bars (n=4).

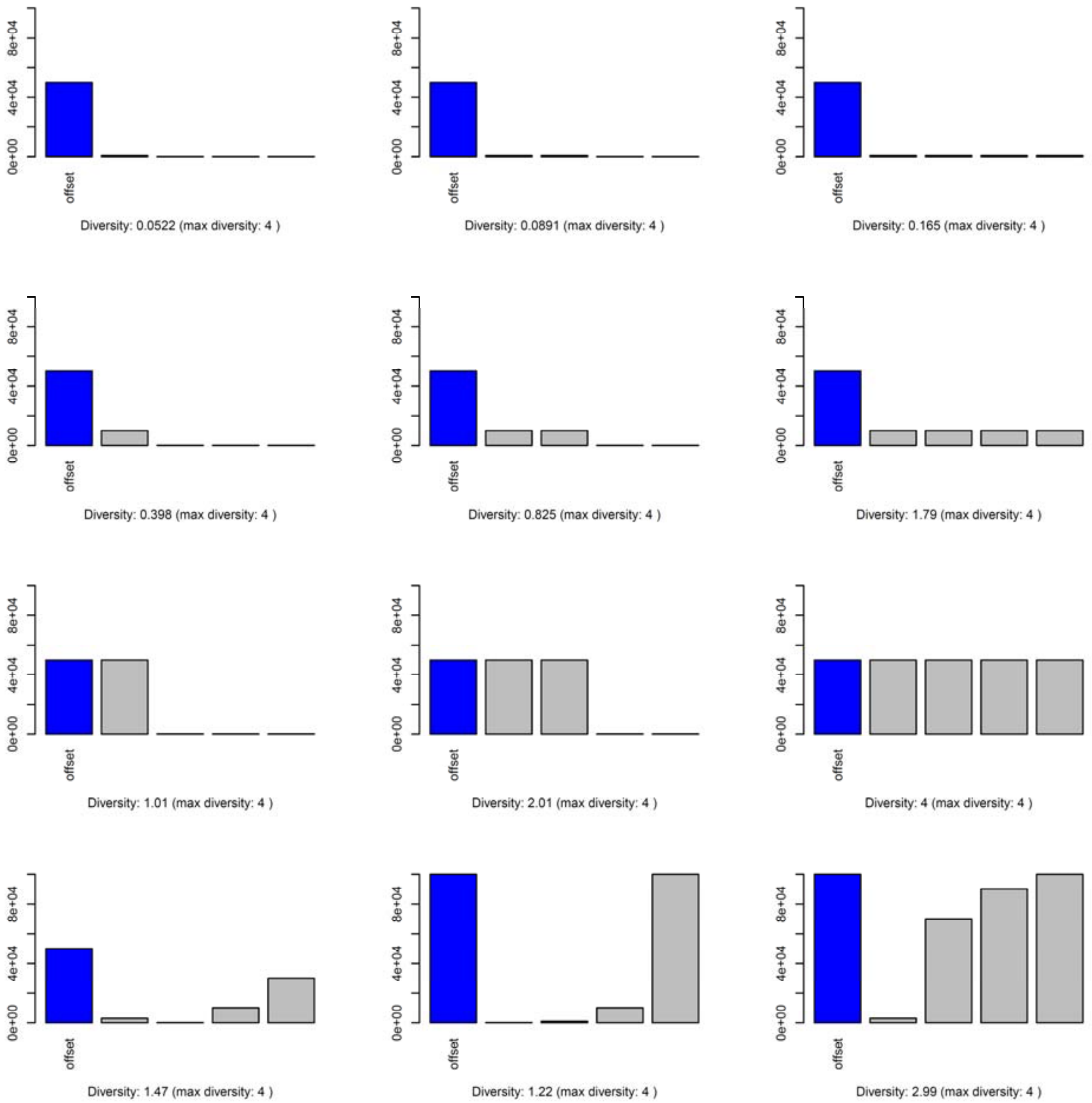

References

1. De Bruin E, Loeber JG, Meijer A, Castillo GM, Cepeda MLG, Torres-Sepúlveda MR, et al. Evolution of an influenza pandemic in 13 countries from 5 continents monitored by protein microarray from neonatal screening bloodspots. J Clin Virol. 2014;61: 74–80. doi:10.1016/j.jcv.2014.06.020
2. Jost L. Entropy and diversity. Oikos. 2006;113: 363–375. doi:10.1111/j.2006.0030-1299.14714.x
